# Associations of 24 h urinary excretions of α- and γ-carboxyethyl hydroxychroman with plasma α- and γ-tocopherol and dietary vitamin E intake in older adults: the Lifelines-MINUTHE Study

**DOI:** 10.1007/s00394-022-02918-8

**Published:** 2022-06-19

**Authors:** Yinjie Zhu, Jan Frank, Ineke J. Riphagen, Isidor Minović, Michel J. Vos, Manfred L. Eggersdorfer, Gerjan J. Navis, Stephan J. L. Bakker

**Affiliations:** 1 Department of Internal Medicine, Division of Nephrology, University of Groningen, University Medical Center Groningen, Groningen, The Netherlands; 2 Department of Food Biofunctionality (140B), Institute of Nutritional Sciences, University of Hohenheim, Stuttgart, Germany; 3 Department of Laboratory Medicine, University of Groningen, University Medical Center Groningen, Groningen, The Netherlands; 4 Certe Medical Diagnostics and Advice, Medical Center Leeuwarden, Leeuwarden, The Netherlands; 5 DSM Nutritional Products, Kaiseraugst, Switzerland

**Keywords:** Vitamin E intake, Tocopherol, CEHC, 24 h urinary excretion, CEHC/creatinine ratio

## Abstract

**Background:**

Urinary metabolites of vitamin E, i.e., α- and γ-carboxyethyl hydroxychroman (α- and γ-CEHC), have gained increasing attention and have been proposed as novel biomarkers of vitamin E intake and status. However, there are insufficient data on the relationship of plasma α-tocopherol and γ-tocopherol and dietary vitamin E intake with 24 h urinary excretions of α- and γ-CEHC.

**Objectives:**

We aimed to (1) investigate the associations of urinary α- and γ-CEHC/creatinine ratios and 24 h urinary excretions of α- and γ-CEHC with plasma α- and γ-tocopherol, respectively; (2) investigate the associations of urinary α- and γ-CEHC/creatinine ratios and 24 h urinary excretions of α- and γ-CEHC with dietary vitamin E intake, and we hypothesize that 24 h urinary excretions of α- and γ-CEHC will better correlate with vitamin E intake than urinary α- and γ-CEHC/creatinine ratios.

**Design:**

24 h Urine and plasma samples were collected from 1519 participants (60–75 years, male: 50%) included in the Lifelines-MINUTHE Study for the assessments of urinary α- and γ-CEHC/creatinine ratios and 24 h urinary excretions of α- and γ-CEHC, and plasma α- and γ-tocopherol. Among those participants, dietary vitamin E intake data from 387 participants were available from an externally validated Flower-Food Frequency Questionnaire (FFQ). The associations of plasma α- and γ-tocopherol, dietary vitamin E intake, with urinary α- and γ-CEHC were assessed using multivariate linear regressions.

**Results:**

24 h Urinary excretion of α-CEHC (median (IQR): 0.9 (0.3–2.4) µmol) was less than that of γ-CEHC (median (IQR): 1.5 (0.5–3.5) µmol). After adjustment for covariates, we found that 24 h urinary α-CEHC excretion and urinary α-CEHC/creatinine ratio were both positively associated with plasma α-tocopherol (std.beta: 0.06, *p* = 0.02; std.beta: 0.06, *p* = 0.01, respectively). Furthermore, the sum of 24 h urinary α- and γ-CEHC excretions was positively associated with dietary vitamin E intake (std.beta: 0.08; *p* = 0.03), whereas there was no relation between urinary α- and γ-CEHC/creatinine ratios and vitamin E intake. No association was observed neither between plasma α- and γ-tocopherol and dietary vitamin E intake, nor between urinary γ-CEHC and plasma γ-tocopherol.

**Conclusion:**

Our study confirmed our hypothesis that 24 h urinary α- and γ-CEHC excretions would be a better marker for dietary vitamin E intake than urinary α- and γ-CEHC/creatinine ratios. Considering that both 24 h urinary α- and γ-CEHC excretions and α- and γ-CEHC/creatinine ratios were also associated with plasma α-tocopherol status, we suggest that 24 h urinary α- and γ-CEHC excretions could be used to assess overall vitamin E status.

**Supplementary Information:**

The online version contains supplementary material available at 10.1007/s00394-022-02918-8.

## Introduction

Vitamin E is the generic term for a group of lipid-soluble molecules consisting of four tocopherols (α, β, γ, δ) and four tocotrienols (α, β, γ, δ). Major dietary sources of vitamin E are nuts and edible vegetable oils, and the proportions of the four tocopherols vary according to the food source, with α-tocopherol and γ-tocopherol as most abundant representatives [1, 2].

Although plasma α-tocopherol has been widely used to assess vitamin E status [3], there is no consistent association found between the plasma α-tocopherol and dietary α-tocopherol intake [4–6]. The adjusted correlations between dietary α-tocopherol intake and plasma/serum α-tocopherol have been reported to be positive [4, 6], negative [7], or null [5]. This inconsistency could be explained by that plasma α-tocopherol is highly dependent on serum lipids and therefore is also dependent on hyperlipidemia [8]. Yet, more evidence is needed to solidify the relation between plasma α-tocopherol and dietary α-tocopherol intake.

α-Carboxyethyl hydroxychroman (α-CEHC) is a side chain-shortened metabolite of α-tocopherol and excreted with the urine [2] and has been proposed as a biomarker of dietary α-tocopherol intake. Among 76 free-living healthy Japanese women (18–22 years), the intake of α-tocopherol was significantly related to urinary α-CEHC excretion (*r* = 0.29, *p* = 0.01) [9]. Another study in 233 adults (33.3 ± 12.5 years) observed that urinary α-CEHC excretion was significantly correlated with α-tocopherol intake (*r* = 0.39, *p* = 0.001) [4]. Studies have also found that urinary α-CEHC excretion responded to both α-tocopherol supplementation [9, 10] and hazelnuts consumption [11], was correlated to usual α-tocopherol intake [4], and could be a biomarker to assess α-tocopherol status [4, 12]. Still, there is insufficient evidence available on the relationship of urinary α-CEHC excretion with dietary α-tocopherol intake and plasma α-tocopherol, since previous studies have restricted their populations to a very small size [4, 9–12], a single gender [9], or clinical settings [12]. More importantly, studies have rarely investigated the 24 h urinary excretion of α-CEHC; instead, they used urinary concentration of α-CEHC from spot urine or urinary α-CEHC normalized for creatinine due to the unavailability of 24 h urine collection, which induced methodological limitation in assessment of intake [13].

In the present study, we investigated the relationships between the urinary excretion of vitamin E metabolites α- and γ-CEHC, plasma vitamin E status (i.e., α-tocopherol, γ-tocopherol), and dietary vitamin E intake in a general population. Furthermore, we compared the utilization of 24 h urinary excretions of α- and γ-CEHC and urinary α- and γ-CEHC/creatinine ratios in the assessment vitamin E status. We hypothesized that 24 h urinary α- and γ-CEHC excretions will correlate with vitamin E intake.

## Methods

### Study design and population

The Lifelines Cohort Study is an ongoing observational three-generation population-based follow-up study that aims to investigate the health and health-related behaviors in the process of aging. Between 2006 and 2013, a total of 167,729 residents from Northern Netherlands were included to form a cohort that is representative of the Dutch general population. A detailed description of the Lifelines Cohort Study can be found elsewhere [14, 15]. In short, the first group of participants was recruited from their local general practitioners. Then, participants could indicate whether their family members were interested, as well. Individuals who were also willing to participate in the study could register via an online self-registration. People with severe psychiatric or physical illness, with limited life expectancy (< 5 years), and those with insufficient knowledge of the Dutch language were not eligible and excluded from the study. Participants were physically examined and various biological materials, including plasma, serum, and 24 h urine, were collected and stored in the Lifelines Biobank. Furthermore, adult participants (≥ 18 years) were asked to complete several questionnaires regarding various aspects, such as demographics, socioeconomic condition, and lifestyle behaviors. All participants gave their informed consent for inclusion before they participated in the study. The study was conducted in accordance with the Declaration of Helsinki, and the protocol was approved by the Medical ethical committee of the University Medical Center Groningen Institutional Review Board, The Netherlands (METc approval number 2007/152).

The MINUTHE (MIcroNUTrients and Health inequalities in Elderly) study is a sub-cohort study from the Lifelines Biobank designed to investigate the interrelationship of socioeconomic status (SES), nutritional status, and health inequalities among older adults. For the MINUTHE study, 1605 participants aged between 60 and 75 years, who had available plasma and 24 h urine samples, were selected from the Lifelines Biobank. Since education is more differentiating than income in the Dutch population [16], the classification of SES was based on education attainment. Low SES was defined as at maximum primary school or completed lower vocational or secondary schooling. High SES was defined as completed higher vocational schooling or university education [17]. The selection was performed at both extremes of the SES distribution while taking into account gender balance, so to create four equally sized groups: 400 men and 403 women with low SES, and 402 men and 400 women with high SES. Participants with missing data on urinary α-CEHC or γ-CEHC excretion were eliminated before analysis leaving 1519 participants in the current study (Supplementary figure S1).

### Laboratory methods

Whole blood samples were drawn in the morning between 8:00 and 10:00 am after a period of overnight fasting at baseline and stored at -80 °C at the Department of Laboratory Medicine of the University Medical Center Groningen, The Netherlands, until the time of analysis. Plasma concentrations of total cholesterol (TC) and high-density lipoprotein cholesterol (HDL-C) were measured with an enzymatic colorimetric method, while low-density lipoprotein cholesterol (LDL-C) was measured with an enzymatic method and total triglycerides (TGL) with a colorimetric UV method, all on a Roche Modular P chemistry analyzer (Roche, Basel, Switzerland). Plasma α-tocopherol and γ-tocopherol were measured and separated by liquid chromatography using a Luna Phenyl-Hexyl column (Phenomenex, Utrecht, The Netherlands). Solvents used were 2 mM ammonium acetate dissolved in water with a 0.1% addition of formic acid and 10 mM ammonium acetate dissolved in ethanol/methanol mixture with a 0.1% addition of formic acid. Separation was followed by detection using a triple-quad MS/MS system using deuterated internal standards [α-tocopherol-d6 (IsoSciences, Ambler, AK, USA) and γ-tocopherol-d4 (Toronto Research Chemicals Inc, North York, ON, Canada)]. Serum α-tocopherol inadequacy has been set at < 30 μmol/L [3]. 25-Hydroxyvitamin D were measured using validated in-house liquid chromatography–tandem mass spectrometry (LC–MS/MS) assays, which had coefficients of variation of < 14.1%.

24 h Urine samples were collected at baselines and stored at -80 °C until analysis. Prior to extraction of vitamin E metabolites α- and γ-CEHC (Supplementary figure S2), urine samples were enzymatically treated with beta-glucuronidase (from helix pomatia Sigma Aldrich, Steinheim, Germany). Therefore, to 200 µL urine, 12.5 µL ascorbic acid (0.1 mg/mL) and 25 µL beta-glucuronidase (3 mg per 100 µL 0.1 M acetate buffer) were added and incubated for 2 h at 37 °C and 300 rpm. After enzyme treatment, samples were cooled on ice and acidified with 50 µL acetic acid. Extraction was performed twice with 1 mL hexane:dichloromethane (50:50 containing 0.1% BHT). Organic upper layers were combined and evaporated to dryness under vacuum. Samples were re-suspended in 50 µL acetonitrile:water (10:90) and 40 µL were injected into the high-performance liquid chromatography (HPLC) system. The separation was performed on a C18 column (AQ, 5 µm, 250 × 4,6 mm, Dr. Maisch, Ammerbuch, Germany) and a Jasco HPLC system consisting of a AS-2059 Plus autosampler, PU-2080 Plus pump, and LG-2080–02 gradient unit; a three-line degasser (Jasco, Groß-Umstadt, Germany) equipped with an electrochemical detector SA Model 5600A CoulArray Detector (ESA Inc., Chelmsford, MA, USA). The electrode potentials were set to 250, 325, 400, and 475 mV. The mobile phase consisted of 50 mM ammonium acetate and acetonitrile (80:20), and the flow rate was set to 1.5 mL/min. The column and electrodes were maintained at 40 °C and the autosampler cooled to 4 °C. Quantification was carried out against external authentic standard curves (Cayman Chemicals, Ann Arbor, Michigan, US) (Supplementary figure S3). The method for the quantification of α- and γ-CEHC was precise (≤ 6.8%CV at high (20 µmol/L) and ≤ 9.1%CV at low concentrations (0.1 µmol/L)), accurate (≤ 3.6%bias at high and < 18.4%bias at low concentrations), and a recovery of 96.4% at high and 108.3% at low concentrations of α-CEHC and 99.2% at high and 108.3% at low concentrations of γ-CEHC. The lower limit of quantification (LLOQ) was 0.9 nmol/L for α-CEHC and 0.1 nmol/L for γ-CEHC. Urinary α- and γ-CEHC concentrations were presented as µmol/L and normalized for urinary creatinine as µmol/mmol creatinine, and subsequently, the 24 h urinary α- and γ-CEHC excretions were calculated by multiplying urinary α- and γ-CEHC concentrations with the recorded 24 h urine volume.

### Dietary intake

Habitual dietary fat and energy intakes were estimated from a semi-quantitative self-reported food frequency questionnaire (FFQ) using the 2011 Dutch food composition database (NEVO) [18]. The FFQ was developed and validated by Wageningen University to assess the intake of 110 food items over the last month [19, 20]. Daily dietary vitamin E intake was estimated from the Petal 3 FFQ of the external validated “Flower-FFQ” to derive habitual nutrients intake using NEVO, as well. The Petal 3 FFQ consists of 64 items to assess the intake of retinol equivalents, vitamin C, vitamin E, and dietary fiber (focusing on, e.g., vegetables, fruits, bread, grains (incl. pasta and rice), and fats and oils). A more detailed description of the development and validation of the “Flower-FFQ” can be found elsewhere [21]. In this study, vitamin E intake data were available for 387 participants. The dietary vitamin E intake estimated from the “Flower-FFQ” was total vitamin E intake adjusted for biological activity, and the intake of specific types of tocopherol was not available. The total vitamin E intake was calculated as mg α-tocopherol equivalents based on: mg alpha-tocopherol + mg beta-tocopherol $\times$ 0.40 + mg gamma-tocopherol $\times$ 0.10 + mg delta-tocopherol $\times$ 0.01 [22]. However, the cut-off of adequate vitamin E intake was derived from European Food Safety Authority (EFSA): an inadequate intake for α-tocopherol was set at ≤ 13 mg/day for men and ≤ 11 mg/day for women [23], because currently there is no cut-off available for total vitamin E intake.

### Other covariates

Anthropometric measurements and blood pressure (BP) were measured by well-trained staff. Anthropometric measurements were taken without shoes. Body weight was measured to 0.1 kg on a SECA 761 scale (Seca GmbH, Hamburg, Germany); height was measured to 0.5 cm using the Frankfort Plane position by the SECA 222 stadiometer (Seca GmbH, Hamburg, Germany) [24]. Body mass index (BMI) was calculated as body weight (kg) divided by height squared (m^2^). The BMI was additionally categorized into underweight (BMI < 20 kg/m^2^), normal (20 ≤ BMI < 30 kg/m^2^), and overweight and obese (BMI ≥ 30 kg/m^2^) according to the fact sheet provided for older adults by the Netherlands nutrition center [25]. Body surface area (BSA) was calculated according the DuBois Formula: BSA (m^2^) = $\frac{\sqrt{\mathrm{Height }\left(\mathrm{cm}\right)\times \mathrm{Weight }(\mathrm{kg})}}{3600}$. Blood pressure was measured with a Dynamap PRO 100V2 (GE Healthcare, Freiburg, Germany); systolic and diastolic blood pressures were measured ten times within 10 min, and each of the average values of the last three readings was used as blood pressure parameters [24].

Smoking status and vitamin supplement use were derived from self-administrated questionnaires. Smoking status was categorized into never, former, and current smoker. Supplementation use was categorized binarily into the use of any vitamin supplements or none. The use of lipid-lowering medication was included as a binary variable reported from participants and subsequently recoded into the anatomical therapeutic chemical (ACT) code (C10A and C10B), as lipid-lowering medication may directly impact plasma tocopherol status [26].

### Statistical analyses

Participants’ characteristics were presented for all, and continuous data are presented as the mean ± standard deviation (SD) when normally distributed or as the median and interquartile range (IQR) for non-normally distributed variables. Categorical variables are presented as percentages. Between-group differences were assessed by ANOVA, Kruskal–Wallis, and Chi-square two-sided tests for normally distributed continuous data, non-normally distributed continuous data, and categorical data, respectively. Pairwise Pearson correlations were computed among 24 h urinary α- and γ-CEHC excretions, the sum of 24 h urinary α- and γ-CEHC excretions, urinary α- and γ-CEHC/creatinine ratios, plasma α- and γ-tocopherol, and dietary vitamin E intake. The *p* values of the correlation coefficient were adjusted for multiple tests.

The cubic roots of 24 h urinary α- and γ-CEHC excretions and urinary α- and γ-CEHC/creatinine ratios, and log-transformed dietary vitamin E intake were used in the regression models to increase the normality. In addition, plasma α- and γ-tocopherol were normalized for plasma total lipids by dividing tocopherol concentrations by the total lipids. We applied non-linearity test using the R-package “rms” to assess the relationship between urinary vitamin E metabolites, plasma tocopherols, and dietary vitamin E intake. Since we did not observe any non-linearity among them, linear regression and logistic regression models were built to assess those relationships. All linear regression models were presented as standard coefficient (95% CI).

First, several linear regression models were built to assess the associations of 24 h urinary α- and γ-CEHC excretions, the sum of α- and γ-CEHC urinary excretions, and urinary α- and γ-CEHC/creatinine ratios with plasma α-tocopherol and γ-tocopherol, respectively. Univariate and multivariate models adjusted for age, sex, BSA, TC, 25-hydroxyvitamin D, use of lipid-lowering medication, smoking status, and SES were shown. Second, linear regression models were built to assess the associations of 24 h urinary α- and γ-CEHC excretions, the sum of α- and γ-CEHC urinary excretions, and urinary α- and γ-CEHC/creatinine ratios with dietary vitamin E intake. Univariate and multivariate models adjusted for age, sex, BSA, supplementation use, dietary lipids intake, smoking status, and SES were shown. Then, we assessed the relationships of plasma α- and γ-tocopherol with dietary vitamin E intake also by using univariate model and multivariate models adjusted for age, sex, BSA, supplementation use, dietary lipids intake, smoking status, and SES.

Potential interactions were also explored by fitting interaction terms between urinary α- and γ-CEHC/creatinine ratios, 24 h urinary α- and γ-CEHC excretions as well as by age, sex, BSA, smoking status, SES, and 25-hydroxyvitamin D with each vitamin E urinary metabolites in associations with plasma α- and γ-tocopherol or vitamin E intake. Missing information on covariates in the association between CEHCs and tocopherols (BSA: 0.1%, smoking status: 0.8%, 25-hydroxyvitamin D: 6.3%) was imputed using ten folded multiple imputation using the mice package in R.

All analyses were carried out using RStudio (version 1.1.463; RStudio Inc., Boston, USA).

## Results

Participants’ characteristics are shown for all in Table 1. As a result of the design of the Lifelines-MINUTHE Study, participants were older adults with an average age of 66 ± 4 years, and half of them were male and had a low education level. Among all participants, 20.6% were overweight or obese, and more than half were former smokers. Mean (SD) concentrations of plasma α- and γ-tocopherol were 34.3 ± 7.9 µmol/L and 1.7 ± 0.7 µmol/L, respectively; around 30% of the participants had inadequate plasma α-tocopherol concentrations. Plasma concentration of α-tocopherol was higher than the concentration of γ-tocopherol, while 24 h urinary excretion of α-CEHC (median (IQR): 0.9 (0.3–2.4) µmol) was lower than the excretion of γ-CEHC (median (IQR): 1.5 (0.5–3.5) µmol). Regarding the dietary intake, participants consumed 1897.5 ± 515.6 kcal and 74.2 ± 26.2 g fat per day, and the median (IQR) dietary vitamin E intake of the 387 participants with data on vitamin E intake was 11.0 (8.3–14.5) mg/day, and 56.6% of them had inadequate vitamin E intake (Table 1).

**Table 1:** Participants’ characteristics

	*N* = 1519
Age,	66 ± 4
Sex, male%	50.0
BMI	27.0 ± 4.2
Underweight	2.0
Normal	77.4
Overweight and Obese	20.6
BSA, m^2^	1.9 ± 0.4
SES, low%	50.0
Supplementation use%	14.6
Lipid-lowering medication%	24.7
Smoking status, %	
Current	12.1
Former	53.8
Never	34.1
Serum	
α-Tocopherol, µmol/L	34.3 ± 7.9
Inadequacy,%	29.3
γ-Tocopherol, µmol/L	1.7 ± 0.7
α-Tocopherol/total lipid, µmol/mmol	5.2 ± 0.7
γ-Tocopherol/total lipid, µmol/mmol	0.2 (0.2–0.3)
25-Hydroxyvitamin D	63.2 ± 22.2
Total lipids, mmol/L	6.7 ± 1.3
TC, mmol/L	5.4 ± 1.1
HDL-C, mmol/L	1.5 ± 0.4
LDL-C, mmol/L	3.5 ± 1.0
TGL, mmol/L	1.1 (0.8–1.5)
Urine	
α-CEHC, µmol/24 h	0.9 (0.3–2.4)
γ-CEHC, µmol/24 h	1.5 (0.5–3.5)
α-CEHC, µmol/L	0.5 (0.2–1.2)
γ-CEHC, µmol/L	0.9 (0.3–1.9)
α-CEHC/creatinine, µmol/mmol	0.08 (0.03–0.2)
γ-CEHC/creatinine, µmol/mmol	0.13 (0.05–0.3)
Dietary intake	
Vitamin E intake, mg/day (*n* = 387)	11.0 (8.3–14.5)
Inadequate intake, %	56.6
Total energy intake, kcal/day	1897.5 ± 515.6
Total fat intake, g/day	74.2 ± 26.2

Missing data were low (BSA: 0.1%, smoking status: 0.8%, 25-hydroxyvitamin D: 6.3%). Serum α-tocopherol inadequacy: < 30 μmol/L; inadequate intake of Vitamin E: ≤ 13 mg/day for men and ≤ 11 mg/day for women

*BMI* body mass index, *BSA* body surface area, *SES* socioeconomic status, *TC* total cholesterol, *HDL-C* high-density lipoprotein, *LDL-C* low-density lipoprotein, *CEHC* carboxyethyl hydroxychroman

Pearson correlation tests showed that dietary vitamin E intake was positively correlated with 24 h urinary excretion of α-CEHC and the sum of 24 h urinary excretions of α- and γ-CEHC, with adjusted correlation coefficients of 0.1 and 0.13 (*p* < 0.01 for both). Moreover, plasma α-tocopherol level was positively corelated with urinary α- and γ-CEHC in both molar creatinine ratio and 24 h excretion forms, with adjusted correlation coefficients between 0.06 and 0.08 (*p* < 0.05) (Supplementary table S1).

Table 2 presents the relation between plasma tocopherols and urinary CEHCs. A higher 24 h urinary excretion of α-CEHC and the sum of α- and γ-CEHC were both positively associated with higher plasma α-tocopherol (std.beta (95%CI): 0.06 (0.01–0.11), *p* = 0.02; 0.07 (0.02–0.12), *p* = 0.007, respectively), but not with plasma γ-tocopherol (Table 2, multivariate). Similar results were also found for urinary α- and γ-CEHC/creatinine ratios with plasma α- and γ-tocopherol (Table 2, multivariate). In all models, plasma total cholesterol and the use of lipid-lowering medication were negatively and positively associated with plasma α- and γ-tocopherol, respectively, while 25-hydroxyvitamin D was positively and negatively associated with plasma α- and γ-tocopherol, respectively.

**Table 2:** Univariable and multivariable associations of urinary α- and γ-CEHC normalized for creatinine and 24 h urinary α- and γ-CEHC excretions with plasma α- and γ-tocopherol levels separately

	Plasma α-tocopherol level (µmol/mmol)^b^		Plasma γ-tocopherol level (µmol/mmol)^b^	
	Standardized beta (95% CI)	*P*	Standardized beta (95% CI)	*P*
**Univariable**				
α-CEHC (µmol/mmol)^a^	0.07 (0.02, 0.12)	0.01	−0.05 (−0.10, 0.00)	0.07
**Univariable**				
γ-CEHC (µmol/mmol)^a^	0.06 (0.02, 0.12)	0.01	0.04 (−0.01, 0.10)	0.1
**Multivariable**				
α-CEHC (µmol/mmol)^a^	0.06 (0.01, 0.11)	0.01	−0.04 (−0.09, 0.01)	0.1
25-hydroxyvitamin D	0.09 (0.04, 0.14)	< 0.001	−0.03 (−0.08, 0.02)	0.1
γ-CEHC (µmol/mmol)^a^	0.04 (−0.01,0.10)	0.1	0.04 (−0.01, 0.09)	0.09
Age	0.01 (−0.04, 0.06)	0.7	−0.07 (−0.12,−0.01)	0.01
Male	−0.01 (−0.13, 0.12)	0.8	−0.12 (−0.24, 0.01)	0.06
BSA	−0.08 (−0.14,−0.02)	0.01	0.03 (−0.03, 0.10)	0.3
Smoking				
Former	0.05 (−0.06, 0.16)	0.5	0.10 (−0.01, 0.21)	0.04
Current	−0.13 (−0.30,−0.04)	0.1	0.03 (−0.14, 0.19)	0.6
Never	Ref		Ref	
SES	0.10 (0.00, 0.21)	0.05	−0.02 (−0.12, 0.09)	0.7
Total cholesterol	−0.16 (−0.22,−0.10)	< 0.001	−0.14 (−0.20,−0.08)	< 0.001
Lipid-lowering medication	0.25 (0.11, 0.38)	< 0.001	0.20 (0.07, 0.34)	0.007
**Univariable**				
α-CEHC (µmol/24 h)	0.06 (0.01, 0.11)	0.02	−0.04 (−0.09, 0.01)	0.1
**Univariable**				
γ-CEHC (µmol/24 h)	0.07 (0.02, 0.12)	0.008	0.05 (0.01, 0.10)	0.08
**Multivariable**				
α-CEHC (µmol/24 h)	0.06 (0.01, 0.11)	0.02	−0.04 (−0.09, 0.01)	0.1
25-Hydroxyvitamin D	0.11 (0.06, 0.16)	< 0.001	−0.02 (−0.07, 0.03)	0.1
γ-CEHC (µmol/24 h)	0.05 (−0.01,0.10)	0.06	0.04 (−0.01, 0.09)	0.1
Age	0.01 (−0.04, 0.06)	0.8	−0.07 (−0.12,−0.02)	0.01
Male	−0.03 (−0.16, 0.09)	0.6	−0.12 (−0.24, 0.01)	0.06
BSA	−0.09 (−0.15, -0.02)	0.004	0.03 (−0.04, 0.09)	0.3
Smoking				
Former	0.05 (−0.06, 0.16)	0.4	0.11 (0.01, 0.22)	0.04
Current	−0.13 (−0.30, 0.04)	0.1	0.02 (−0.15, 0.19)	0.6
Never	Ref		Ref	
SES	0.11 (0.01, 0.21)	0.04	−0.04 (−0.14, 0.07)	0.7
Total cholesterol	−0.16 (−0.22,−0.10)	< 0.001	−0.13 (−0.19,−0.07)	< 0.001
Lipid-lowering medication	0.24 (0.11–0.38)	< 0.001	0.19 (0.06–0.33)	0.007
Univariable				
α + γ-CEHC (µmol/24 h)	0.08 (0.03, 0.13)	0.002	0.0001 (−0.05, 0.05)	0.8
Multivariable				
α + γ-CEHC (µmol/24 h)	0.07 (0.02, 0.12)	0.007	0.002 (−0.05, 0.05)	0.9
25-Hydroxyvitamin D	0.10 (0.05, 0.15)	< 0.001	−0.04 (−0.09, 0.01)	0.09
Age	0.01 (−0.04, 0.06)	0.7	−0.07 (−0.12,−0.02)	0.01
Male	−0.03 (−0.16, 0.09)	0.6	−0.12 (−0.24, 0.01)	0.06
BSA	−0.09 (−0.15,−0.03)	0.005	0.03 (−0.03, 0.09)	0.3
Smoking				
Former	0.03 (−0.08, 0.14)	0.5	0.12 (0.01, 0.23)	0.04
Current	−0.14 (−0.31, 0.03)	0.1	0.04 (−0.12, 0.21)	0.6
Never	Ref		Ref	
SES	0.10 (0.01,0.20)	0.05	−0.02 (−0.12, 0.08)	0.7
TC	−0.16 (−0.22,−0.10)	< 0.001	−0.14 (−0.20,−0.09)	< 0.001
Lipid-lowering medication	0.25 (0.12–0.39)	< 0.001	0.19 (0.05, 0.32)	0.007

*CEHC* carboxyethyl hydroxychroman, *BSA* body surface area, *SES* socioeconomic status, *TC* total cholesterol

^a^Normalized for urinary creatinine

^b^Normalized for total plasma lipids

In terms of dietary vitamin E intake, the association between 24 h α-CEHC urinary excretion and vitamin E intake remained borderline significant after adjustment for covariates (std.beta (95%CI): 0.07 (0.01–0.15), *p* = 0.05) (Table 3). More importantly, the sum of 24 urinary excretions of α- and γ-CEHC was positively associated with dietary vitamin E intake independent of dietary lipids intake (std.beta (95%CI): 0.08 (0.01–0.16), *p* = 0.03) (Table 3). However, when urinary α- and γ-CEHC were normalized for urinary creatinine, no association was observed with dietary vitamin E intake (Table 4). It is worth mentioning that dietary lipid intake correlated strongly with dietary vitamin E intake in all models (std.beta (95%CI): 0.69 (0.61–0.77), *p* < 0.001 for all; Tables 3, 4). No statistically significant association was observed between plasma tocopherols and dietary vitamin E intake (Supplementary table S2). No interaction was found between any of the covariates tested with vitamin E urinary metabolites.

**Table 3:** Univariable and multivariable associations of 24 h urinary α- and γ-CEHC excretions with vitamin E intake (*n* = 387)

	Vitamin E intake^a^							
	Standardized beta (95% CI)	*P*		Standardized beta (95% CI)	*P*		Standardized beta (95% CI)	*P*
**Univariable**			Univariate			Univariate		
α + γ-CEHC (µmol/24 h)	0.13 (0.03, 0.23)	0.01	α-CEHC (µmol/24 h)	0.10 (0.01, 0.20)	0.04	γ-CEHC (µmol/24 h)	0.09 (−0.01, 0.19)	0.06
**Multivariable**			Multivariate			Multivariate		
α + γ-CEHC (µmol/24 h)	0.08 (0.01, 0.16)	0.03	α-CEHC (µmol/24 h)	0.07 (0.01, 0.15)	0.05	γ-CEHC (µmol/24 h)	0.05 (−0.02, 0.13)	0.2
Age	−0.01 (−0.09, 0.06)	0.7	Age	−0.01 (−0.09,0.06)	0.8	Age	−0.01 (−0.09, 0.06)	0.7
Male	−0.10 (−0.30, 0.09)	0.3	Male	−0.11 (−0.30, 0.09)	0.3	Male	−0.11 (−0.31,0.08)	0.2
Supplementation use	0.08 (−0.11, 0.28)	0.4	Supplementation use	0.09 (−0.10, 0.29)	0.3	Supplementation use	0.09 (−0.11, 0.29)	0.4
BSA	0.05 (−0.05, 0.14)	0.3	BSA	0.05 (−0.05, 0.14)	0.3	BSA	0.05 (−0.04, 0.15)	0.3
Smoking			Smoking			Smoking		
Former	0.06 (−0.11, 0.23)	0.5	Former	0.05 (−0.11, 0.22)	0.5	Former	0.05 (−0.12,0.21)	0.6
Current	−0.07 (−0.32, 0.19)	0.6	Current	−0.08 (−0.33, 0.18)	0.6	Current	−0.07 (−0.32,0.19)	0.6
Never	Ref		Never	Ref		Never	Ref	
Lipids intake	0.69 (0.61, 0.77)	< 0.001	Lipids intake	0.69 (0.61,0.77)	< 0.001	Lipids intake	0.69 (0.61, 0.77)	< 0.001
SES	0.03 (−0.13, 0.18)	0.7	SES	0.02 (−0.14,0.18)	0.8	SES	0.03 (−0.13, 0.19)	0.7

*CEHC* carboxyethyl hydroxychroman, *BSA* body surface area, *SES* socioeconomic status

^a^Log transformed

**Table 4:** Univariable and multivariable associations of urinary α- and γ-CEHC normalized for creatinine with vitamin E intake (*n* = 387)

	Vitamin E intake^a^				
	Standardized beta (95% CI)	*P*		Standardized beta (95% CI)	*P*
**Univariable**			Univariable		
α-CEHC (µmol/mmol)^b^	0.05 (−0.05, 0.15)	0.3	γ-CEHC (µmol/mmol)^b^	0.04 (−0.06, 0.14)	0.4
**Multivariable**			Multivariable		
α-CEHC (µmol/mmol)^b^	0.07 (−0.01, 0.14)	0.07	γ-CEHC (µmol/mmol)^b^	0.05 (−0.03, 0.19)	0.2
Age	−0.01 (−0.09, 0.06)	0.7	Age	−0.02 (−0.09, 0.06)	0.7
Male	−0.09 (−0.29, 0.10)	0.3	Male	−0.10 (−0.30, 0.09)	0.3
Supplementation use	0.09 (−0.10, 0.29)	0.3	Supplementation use	0.09 (−0.11, 0.29)	0.4
BSA	0.05 (−0.04, 0.15)	0.3	BSA	0.06 (−0.04, 0.15)	0.2
Smoking			Smoking		
Former	0.05 (−0.12,0.22)	0.5	Former	0.05 (−0.12, 0.21)	0.6
Current	−0.08 (−0.33, 0.18)	0.5	Current	−0.07 (−0.32, 0.19)	0.6
Never			Never		
Lipids intake	0.69 (0.61, 077)	< 0.001	Lipids intake	0.69 (0.61, 0.77)	< 0.001
SES	0.02 (−0.14, 0.15)	0.8	SES	0.03 (−0.13, 0.19)	0.7

*CEHC* carboxyethyl hydroxychroman, *BSA* body surface area, *SES* socioeconomic status

^a^Log transformed

^b^Normalized for urinary creatinine

## Discussion

In this study, we aimed to explore the relationship of urinary vitamin E metabolites, i.e., α- and γ-CEHC, represented in both 24 h urinary excretions and molar creatinine ratios with plasma tocopherols and dietary vitamin E intake. We found that both 24 h urinary α-CEHC excretion and urinary α-CEHC/creatinine ratio were positively associated with plasma α-tocopherol status, and the sum of 24 h urinary α- and γ-CEHC excretions was positively associated with dietary vitamin E intake, which was in agreement with our hypothesis that 24 h urinary excretions of α- and γ-CEHC might be a better marker for vitamin E intake than urinary α- and γ-CEHC/creatinine ratios.

We included a rather large population to investigate the relation between plasma tocopherols and urinary excretions of CEHC, and found that both 24 h urinary α-CEHC excretion and urinary α-CEHC/creatinine ratio could be an indicator of plasma α-tocopherol status. Assessing plasma α-tocopherol status is of clinical importance, because circulating plasma α-tocopherol has been associated with metabolic syndrome and gallstone disease [27, 28]. Our findings are in agreement with the limited available evidence in the literature, in which Lebold et al*.* reported a strong adjusted correlation of urinary α-CEHC with plasma α-tocopherol [4]. Although plasma α-tocopherol level is often used as a biomarker for vitamin E intake status and was also related to the unhealthy dietary pattern [29], we failed to observe any correlation between plasma tocopherols and vitamin E intake. Our results, to some extent, correspond with previous inconsistent and contradictory results found between plasma α-tocopherol and vitamin E intake [4–7]. A study has suggested that circulating plasma α-tocopherol is correlated with plasma total lipids, a higher level of α-tocopherol does not necessarily represent a higher intake of α-tocopherol [8]. Also, the null relation between plasma tocopherol and dietary vitamin E intake indicated that plasma tocopherol levels were well regulated and held constant in our body. Therefore, it is likely not possible to assess vitamin E intake based solely on plasma tocopherols. However, we cannot neglect the need to find novel biomarkers to objectively assess vitamin E intake, since some clinical interventions with vitamin E have provided beneficial effects, including reduced risk for macular degeneration in the older adults [30], less functional decline in patients with Alzheimer’s disease [31], and the treatment of nonalcoholic steatohepatitis in children, adolescents, and adults without diabetes [32–34].

In the last 2 decades, the urinary vitamin E metabolite α-CEHC has been suggested to be a novel indicator of an adequate vitamin E intake [35]. The physiological pathway from tocopherols to CEHC is well established. Tocopherols are metabolized in the liver by ω-hydroxylation of the terminal methyl group via cytochrome P (CYP) 4F2, followed by a several rounds of β-oxidation that rapidly proceeds to the formation of the final product CEHC. Subsequently, the CEHC are excreted via feces, urine, or skin [23]. Despite the null association between plasma tocopherols and vitamin E intake, we found that the sum of 24 h urinary excretion of α- and γ-CEHC was positively associated with vitamin E intake after adjusting for covariates. On the other hand, we did not observe the same association between urinary α- and γ-CEHC/creatinine ratios and dietary vitamin E intake. This indicates that 24 h urinary excretion of α- and γ-CEHC can better reflect daily dietary vitamin E intake than urinary α- and γ-CEHC/creatinine ratios, as they are both quantitative parameters.

The dietary vitamin E intake assessed in our study included all dietary tocopherols, using a specially designed and externally validated “Flower-FFQ” instead of relying solely on α-tocopherol, which explains why we did not observe statistically significant association between 24 h urinary excretion of α-CEHC or γ-CEHC alone with dietary vitamin E intake in our multivariate models. More specifically, the sum of 24 h urinary excretion of α- and γ-CEHC would better correspond to the dietary vitamin E intake measured in our study, since α- and γ-tocopherols are the two most abundant tocopherols present in plants and in our diet. Thus, it was not surprising that we observed the positively association between the sum of α- and γ-CEHC, not α-CEHC or γ-CEHC individually, and dietary vitamin E intake. Although previous studies have mostly used urinary α- and γ-CEHC concentrations or α- and γ-CEHC normalized for urinary creatinine, due to the unavailability of 24 h urine, some studies have shown that urinary α-CEHC excretion responds to α-tocopherol supplementation in healthy men or/and women [9, 10, 35] as well as hazelnut consumption among older adults [11]. In short, the sum of 24 h urinary excretion of α- and γ-CEHC seemed to be more sensitive to dietary vitamin E intake compared with plasma tocopherols or urinary α- and γ-CEHC/creatinine ratios, indicating its potential to be considered as a novel biomarker for vitamin E intake. Still, more research is required in larger sample sizes to confirm if CEHC could be a valid biomarkers for vitamin E intake, since the coefficient found in our study was small.

Our findings support the current opinion that urinary excretion of CEHC has the potential to be a novel biomarker of dietary vitamin E intake, and meanwhile add evidence in a general population setting for future policymakers to make decisions on the requirements of vitamin E at a population level. In Europe, the current dietary reference values for vitamin E are set as Adequate Intake (AI) for α-tocopherol for all population groups based on observed intakes estimated from dietary surveys in healthy populations with no apparent α-tocopherol deficiency [23]. Unfortunately, the average requirement (AR) and the population reference intake (PRI) for vitamin E and α-tocopherol cannot be derived, because of insufficient data on biomarkers of vitamin E or α-tocopherol intake and status [23]. This is also the reason why the revision of the dietary reference intake of vitamin E has not seen any progress in North America [36]. Our results might increase awareness among nutrition scholars and policy makers, and promote more research into 24 h urinary excretion of CEHC and their relationships with plasma tocopherols and vitamin E intake. As one of the key aspects of malnutrition, micronutrient deficiencies and insufficiencies are quite prevalent even in developed countries [37]. Hence, objective assessments of 24 h urinary excretions of α- and γ-CEHC could be applied to evaluate vitamin E intake status and to validate dietary intake data for future clinical trials.

This study has several strengths. First, we innovatively incorporated a new angle of urinary CEHC excretion and compared the utilization of both 24 h urinary excretions of CEHC and urinary CEHC/creatinine ratios regarding assessing vitamin E intake and status. Second, we assessed the 24 h urinary excretions of α- and γ-CEHC and plasma tocopherols in a relatively large population, compared to relevant literature, which could increase the generalization of our results. Third, the vitamin E intake was assessed by an externally validated and tailored FFQ designed for the Lifelines Cohort Study, and thus, to some extend increased the accuracy and reliability of vitamin E intake data [21]. However, there are some limitations. First, after collection, our plasma and urine samples had been in storage for 3–10 year at -80 °C. Previous studies showed that tocopherols remain stable in plasma samples for 13–15 years [38], and storage of urine aliquots for periods exceeding 10 years appears also to be an acceptable and valid tool for numerous clinical chemistry parameters [39], so the storage time is unlikely to have an impact on our results. Second, the MINUTHE study only incorporated older adults, which limits the generalization to younger age groups. Third, the amount of vitamin E in a supplement could influence the plasma alpha-tocopherol concentrations [9, 10, 40, 41]. Unfortunately, we were unable to investigate this, because we had no information on types of vitamin supplements and on vitamin E content of the vitamin supplements. In total, 15% of participants reported using any type of vitamin supplement and we adjusted for supplement use as a covariate in our models, which did not alter our results substantially. Therefore, we did not expect that supplementation use would impact on our results. Fourth, we were not able to provide data on tocotrienol intake, plasma tocotrienol concentrations, or urinary metabolites of tocotrienol, because of the design and scope of this study. However, we do acknowledge that there is a growing interest in tocotrienols over tocopherols in some disease outcomes, such as Alzheimer disease and cardiovascular disease [42, 43]. More investigations are needed to identify the novel biomarkers of tocotrienol intake. Finally, urine is not the main excretion pathway of CEHC, and feces and skins are other important excretion pathways. We were not able to capture the excretions of CEHC via feces and skins, which could bias the associations found in this study.

In conclusion, our findings highlight the importance of measuring urinary vitamin E metabolites by showing that 24 h urinary α-CEHC excretion could be an indicator of plasma α-tocopherol status, and the sum of 24 h urinary excretions of α- and γ-CEHC could better assess dietary vitamin E intake compared to urinary α- and γ-CEHC/creatinine ratios. More research in a general younger population is needed to pool sufficient evidence for validating 24 h urinary excretions of α- and γ-CEHC as valid biomarkers for vitamin E intake and status.

## Supplementary Information

Below is the link to the electronic supplementary material.

Supplementary file1 (DOCX 268 KB)

## Data Availability

The authors do not have the authority to share the data that support the findings of this study, due to Lifelines data access permissions, but any researchers can apply to use Lifelines data, including the variables used in this investigation. Information about access to Lifelines data is given on their website: (https://www.lifelines.nl/researcher/how-to-apply).
