# Supplementary material for: Associations of 24 h urinary excretions of α- and γ-carboxyethyl hydroxychroman with plasma α- and γ-tocopherol and dietary vitamin E intake in older adults: the Lifelines-MINUTHE Study
Source: Eur J Nutr. 2022 Jun 19;61(7):3755–65. doi: 10.1007/s00394-022-02918-8 (PMC9464128; doi:10.1007/s00394-022-02918-8)
Supplement: Supplementary file 1 — Supplementary file1 (DOCX 268 KB) [file 394_2022_2918_MOESM1_ESM.docx]

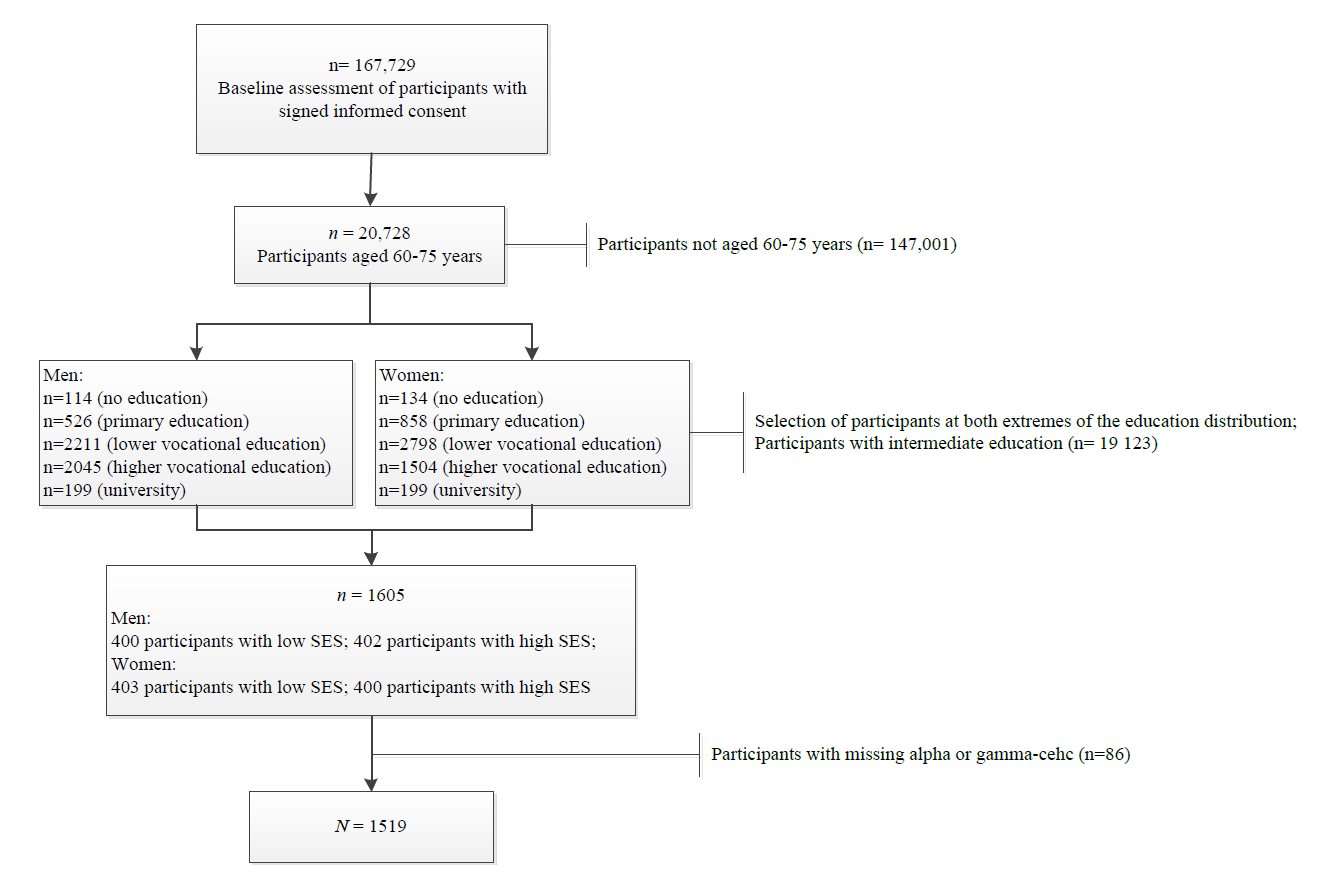


Supplementary figure S1. Study flow chart


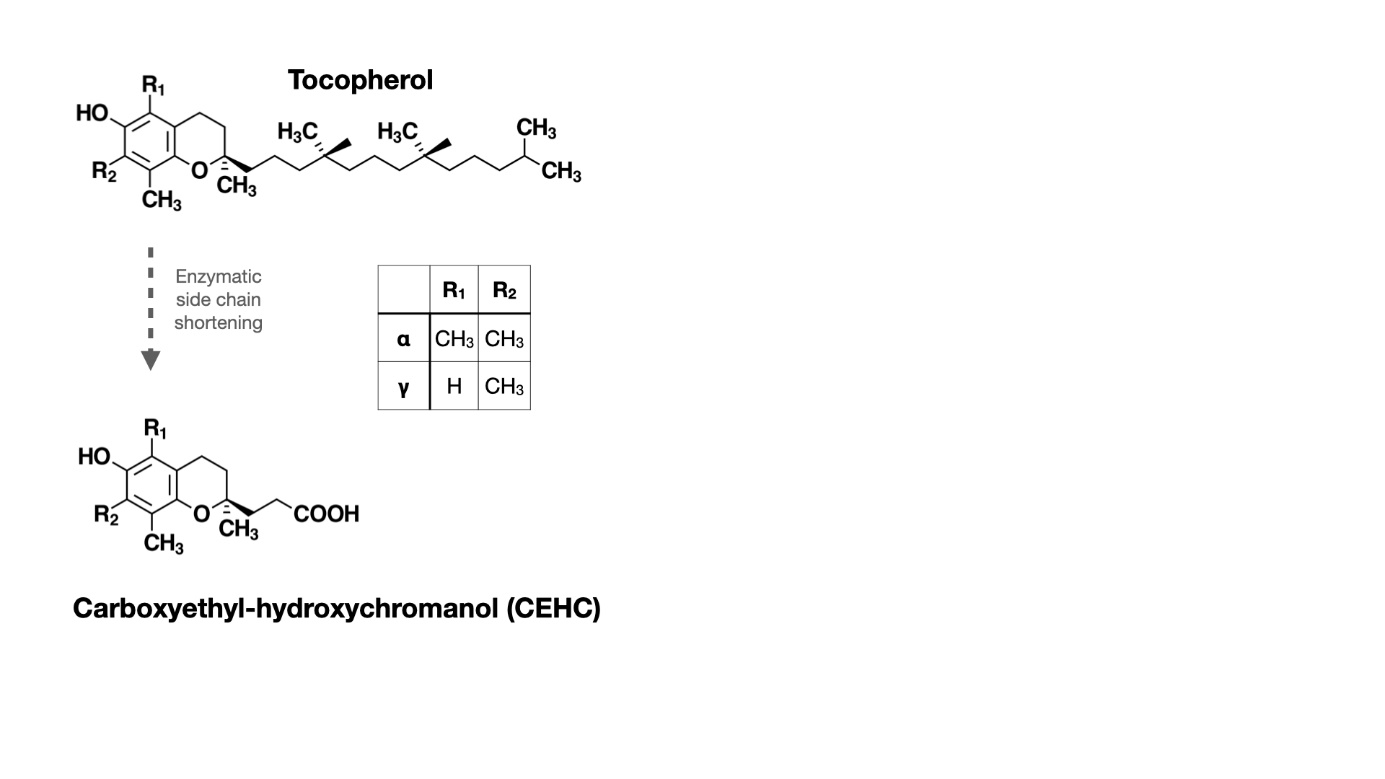


Supplementary figure S2. Chemical structures of tocopherol and CEHC.


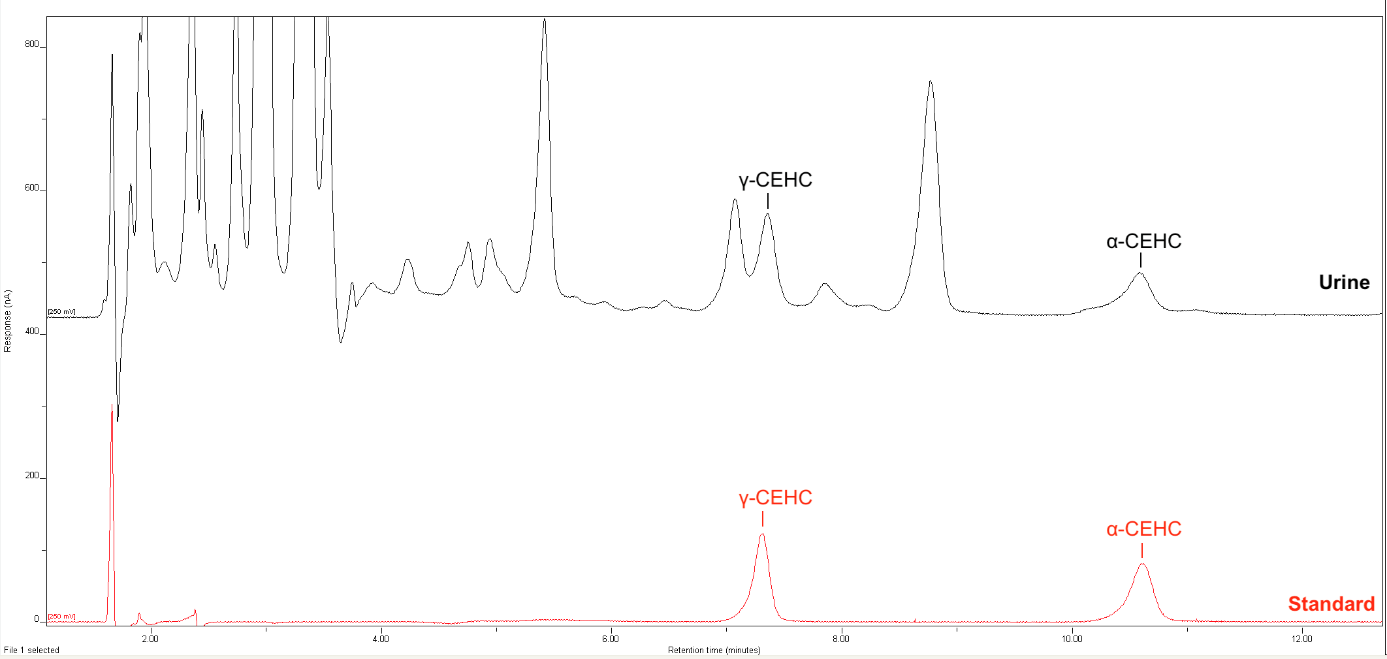


Supplementary figure S3. A representative chromatogram showing a 10 µmol/L reference standard (lower trace, red) and a representative chromatogram from a urine sample (upper trace, black).

Supplementary table S1. Pearson correlation of vitamin E intake, plasma tocopherols and urinary CEHCs adjusted for multiple tests.

|  | α-CEHC (µmol/mmol)^1^ | α-CEHC (µmol/24 h) | γ-CEHC (µmol/mmol)^1^ | γ-CEHC (µmol/24 h) | CEHCs ((µmol/24 h) ) | Daily vitamin E intake (mg/day)^3^ |  |
| --- | --- | --- | --- | --- | --- | --- | --- |
| Plasma α-tocopherol level (µmol/mmol)^2^ | 0.06** | 0.06* | 0.06** | 0.07** | 0.08* | 0.09 |  |
| Plasma γ-tocopherol level (µmol/mmol)^2^ | -0.05* | -0.05 | 0.04 | 0.04 | <0.001 | -0.01 |  |
| Daily vitamin E intake (mg/day)^3^ | 0.05 | 0.1* | 0.04 | 0.09 | 0.13** |  |  |

^1^ normalized for urinary creatinine;^2.^ normalized for total plasma lipids; ^3^log transformed; CEHC: carboxyethyl hydroxychroman; * p < 0.05, ** p < 0.01.

| Supplementary table S2. Associations of plasma α- and γ-tocopherols with dietary vitamin E intake. | | | | | |
| --- | --- | --- | --- | --- | --- |
|  | Vitamin E intake^1^ | | | | |
|  | standardized beta (95%CI) | p |  | standardized beta (95%CI) | p |
| **Univariable** |  |  | **Univariable** |  |  |
| Plasma α-tocopherol level (µmol/mmol)^2^ | 0.09 (-0.01,0.19) | 0.09 | Plasma γ-tocopherol level (µmol/mmol)^2^ | -0.008 (-0.11, 0.09) | 0.9 |
| **Multivariable** |  |  | **Multivariable** |  |  |
| Plasma α-tocopherol level (µmol/mmol)^2^ | 0.03 (-0.05, 0.10) | 0.5 | Plasma γ-tocopherol level (µmol/mmol)^2^ | 0.002 (-0.007, 0.08) | 1.0 |
| Age | -0.01 (-0.09,0.06) | 0.7 | Age | -0.02 (-0.09, 0.06) | 0.7 |
| Male | -0.11 (-0.30,0.09) | 0.3 | Male | -0.11 (-0.31, 0.09) | 0.3 |
| Supplementation use | 0.11 (-0.09, 0.30) | 0.3 | Supplementation use | 0.10 (-0.10, 0.30) | 0.3 |
| BSA | 0.05 (-0.04, 0.15) | 0.3 | BSA | 0.05 (-0.04, 0.15) | 0.3 |
| Smoking |  |  | Smoking |  |  |
| Former | 0.04 (-0.13,0.20) | 0.7 | Former | 0.04 (-0.13, 0.18) | 0.6 |
| Current | -0.07 (-0.32, 0.19) | 0.6 | Current | -0.08 (-0.33, 0.18) | 0.6 |
| Never | Ref |  | Never | Ref |  |
| Lipids intake | 0.69 (0.61, 0.77) | <0.001 | Lipids intake | 0.69 (0.61, 0.77) | <0.001 |
| SES | 0.02 (-0.13,0.18) | 0.8 | SES | 0.02 (-0.13, 0.18) | 0.8 |

^1^ log transformed; ^2^ normalized for total plasma lipids; CEHC: carboxyethyl hydroxychroman, BSA: body surface area, SES: socioeconomic status.

Supplementary table S3. Plasma α- and γ-tocopherols levels stratified by use of lipid-lowering medication.

| On lipid-lowering medication | Yes (n=375) | No (n=1144) | p |
| --- | --- | --- | --- |
| α-tocopherol, µmol/L | 31.2 ± 8.4 | 35.3 ± 7.4 | <0.001 |
| α-tocopherol/total lipid | 5.3 ± 0.8 | 5.1 ± 0.7 | <0.001 |
| γ-tocopherol, µmol/L | 1.7 ± 0.7 | 1.8 ± 0.7 | 0.01 |
| γ-tocopherol/total lipid, µmol/mmol | 0.29 ± 0.1 | 0.25 ± 0.1 | <0.001 |
